# Some Reflections on the Treatment of Rheumatic Manifestations

**Published:** 1901-02

**Authors:** H. A. Richy

**Affiliations:** New York


### SOME REFLECTIONS ON THE TREATMENT OF RHEUMATIC MANIFESTATIONS.

By H. A. RICHY, M. D., New York.

IN THESE days of scientific research the term rheumatism is still an indefinite one, and is applied to a group of painful affections due to different toxic agents. Formerly, it was believed that every form of rheumatism was caused by an excess of uric or lactic acid circulating in the blood. It was further believed that a rheumatic diathesis, inherited or acquired, played an important part in the causation of the disease. Looking at this subject from a modern standpoint it cannot be denied that there are persons who, owing to heredity or more often to an improper mode of living, are particularly predisposed to manifestations of a rheumatic type. Dietary indiscretions, abuse of alcohol, persistent exposure to dampness and cold; all may be predisposing and exciting causes. It is probable that the rheumatic virus, whether it be uric acid, lactic acid, or some other toxin, is generated in consequence of malassimilation and faulty metabolism.

While these theories satisfactorily explain the etiology of a certain number of cases, in others we must search elsewhere for an explanation of their origin. Bacteriology has to a great extent elucidated this question. Although no specific germ has been discovered, the theory of an infectious origin of certain types of rheumatism has been established upon a firm foundation. This is true particularly of those very acute and severe cases of articular rheumatism in which the effusion assumes a purulent character, the pathogenic organism being probably a staphylococcus or streptococcus. It is also interesting to note in this connection that the gonococcus and its toxins are the direct cause of the condition known as gonorrheal rheumatism, the organism having been found in the affected joints and in other fibrous structures.

Rheumatism is a disease of common occurrence in the United States, particularly in the region along the Atlantic coast. Its principal symptom is pain, usually located in one or several articulations. When the disease first attacks some other part of the body, the diagnosis can only be reached by a careful investigation of the patient's history.

In treating rheumatism a limited number of physicians still adhere to the old theory of a lack of alkalinity of the blood, and for this reason employ the salts of potassium, sodium or lithium. With most practitioners, however, salicylic acid, or better, sodium salicylate, seems to be the main standby and is considered as a true specific. Unfortunately, sodium salicylate is by no means a safe drug. Though it promptly suppresses the pain; on the other hand, it gives rise to very disagreeable and even dangerous sequelæ, such as irritation of the stomach, heart weakness, dizziness, ringing in the ears, and the like. To my knowledge, many a case of pericarditis has been brought on by the injudicious use of this remedial agent. In consequence of the objectionable effects just enumerated, when salophen was introduced into the United States, with the recommendation of prominent foreign authorities, I decided to make a trial of it in my next cases. Its superiority over the salicylate of sodium became immediately apparent. The chemical name of salophen is acetylparamidophenol salicylic acid ester. It appears in the form of corlorless crystals, almost tasteless, insoluble in water, but freely soluble in alcohol. It is best administered in wafers, or simply dropped on the tongue. It passes unchanged through the stomach, and in the intestines it separates into salicylic acid and acetyl paramidophenol, the latter having antipyretic and analgesic properties, and supplementing the action of the salicylic acid. The separation is probably very gradual, as the salicylic acid which is formed causes no untoward symptoms whatever, the action of the drug being antirheumatic, antineuralgic and antipyretic at the same time. In severe cases of rheumatism salophen may be given in doses of fifteen grains every two hours for a number of days without provoking complaints of disturbed functions. At times it causes a profuse perspiration, which, however, is more beneficial than weakening.

The following two cases are selected from my records to illustrate the gratifying results produced by salophen:

Case I.—B. R., American, 28 years of age, usual health excellent; no previous history of rheumatism or specific infection. His father and mother are still living; they never suffered from rheumatism; he himself is a moderate drinker. The patient had been ill for a week, and under the care of a physician when I was called to see him. He had some fever and complained of pain in the chest and left arm, with embarrassed breathing. His wife stated that while he was quiet in the day time, at night he was delirious and could hardly be restrained from leaving his bed. Examination revealed the existence of pericarditis; endocarditis was apparently absent. Suspecting that a great deal of the trouble was caused by the large doses of sodium salicylate which the sufferer was taking, I stopped the medicine, and substituted in its place some dry champagne wine, with a substantial nourishment. On my visit the following morning, I was pleased to hear that the patient had spent a quiet night. On the next day the action of the heart continued to improve, and I prescribed salophen in ten grain doses, every three hours, and a tonic. In one week's time the man was fully convalescent, and no relapse occurred to delay a complete cure.

Case II.--Mrs. M. B., Irish-American, 32 years of age; no previous history of rheumatism. She had always been active and strong until December, 1898, when after taking care of two of her children during a long stage of illness, she became much exhausted and unable to attend to her daily duties. On January 1, 1899, she felt very ill and applied to me for help. She complained of sore throat and difficult breathing; her temperature was 103½°. With my finger I could detect the existence of an abscess in the right side of the neck. The patient being in a state of great misery, I called again on her in the evening. Her dyspnea was so severe that I was obliged to make deep scarifications. A dose of fifteen grains of salophen was also given with an happy effect, as the temperature fell off, and the sufferer was made more comfortable. On January 4th, I opened the abscess, and left her with the expectation that she would soon get well. To my surprise the next morning, she began to develop a severe attack of articular rheumatism. Her temperature went up suddenly to 104½°; her tongue became dry, and her lungs congested. She complained of excruciating pains in all the articulations of the left arm and leg. Salophen was then prescribed in doses of fifteen grains, every two hours, the daily dose being limited to ninety grains. This treatment was continued until January 10th, the heart meanwhile remaining undisturbed by the medicine; the stomach in good condition. No pain was felt by the patient unless some of the affected joints were touched. January 11th, her left side was entirely free from pain, while the right arm became suddenly involved, the fever showing some exacerbations. Salophen, which had been reduced to ten grain doses, every three hours, was given again in doses of fifteen grains every two hours. January 12th, the disease assumed a decidedly milder aspect; the temperature went down to 100°; the urine became clear and normal; the tongue cleaner and moist. Severe pain was yet felt at different intervals during the following week, but on January 21st the patient was well enough to dispense with her medical attendance. A simple blood tonic and a few eight grain doses of salophen, three times daily, entirely restored her to health.

During the first months of last winter, in November and December, 1899, an epidemic of influenza raged in New York City. Most of the cases showed above all rheumatic symptoms. As a rule, the disease was not dangerous to life, but its manifestations were rather severe. The attack came on with a chill, followed by fever, pain in the limbs, congestion of the throat and lungs, marked drowsiness; in fact, the complete chain of symptoms which indicate a severe illness of an infectious type. My rule was to put the patient to bed and give him at once a dose of fifteen grains of salophen. A profuse perspiration would invariably set in within a short time, which was aided by warm drinks, and covering the patient with blankets. A few doses of salophen sufficed to complete the cure. The patient was generally able to return to his work after three or four days of rest, the attack having subsided very quickly and leaving no sequelæ beyond a little weakness.

In headaches dependent upon a rheumatic diathesis, in neuralgias due to excess of uric acid in the blood, salophen has been used to advantage by me, after other remedies had failed to give relief.

In a severe case of sciatica which had resisted different drugs, the pain was greatly diminished by several doses of salophen. Unfortunately, the patient, who was a commercial traveler, was compelled to leave New York where he had been detained by his illness, and I am unable to state whether the improvement was permanent or not.

In every case of tonsillitis, in most attacks of sore throat, attended with muscular soreness, salophen may be considered as a specific. To small children I administer the powder mixed with a little sugar, so that the medicine is taken just as readily as a dose of calomel.

In the treatment of scarlet fever, salophen has regularly given encouraging results. It reduces the soreness of the throat to a minimum, and controls the fever without producing depression. The rheumatic manifestations so generally met with in this disease are very slight, if the drug is given at the beginning of the illness. To a child six years of age, three or four grain doses can be administered with entire safety. When there exists a feeling of depression tincture of iron may be added to the treatment.

161 East 46th Street.

